# 

**Published:** 1892-07-16

**Authors:** 


### CONTENTS.

**Hospital Committees, Trained Nurses, and the Public** 257

**Medical History from the Earliest Times.** By E. T. WITHINGTON, M.A., M.B Oxon. XVII.—Early Roman Medicine 259

**Employments for Women.** IV.—Lecturing for the County Councils - 260

**The Attitude of Great Writers towards Disease.** V.—Lord Tennyson - 261

**Everybody's Page** - 262

**Annotations.**—The Hospital Conference—A Royal Consumptive—Dustbins and Cholera - 263

**Notes and News** - 264

**Around the Hospitals** - 265

**Scraps and Gleanings** - 265

**The Student of Medicine.** — Appointments—Vacancies—Pass Lists—Athletics.

**The Practitioners' Mirror and Retrospect—**

MEDICINE.—Thyroid Extract for Myxœdema - 266

OPHTHALMOLOGY.—Interstitial Keratitis - 267

PRACTITIONERS' BOOKSHELF.—Outlines of Insanity, 267; The Harvard Medical Schools - 268

NEW DRUGS, APPLIANCES, AND THINGS MEDICAL.—"St. Bede" Disinfectant—The Reliance Medicine or Liquid Dropper—Triticumina Wholemeal Bread—Kops' Ale - 268

**The Institutional Workshop—**

WITHIN THE HOSPITALS. V.—Addenbrooke's Hospital, Cambridge - 269

INSTITUTION LITERATURE.—Asylums of the World. III.—Administration and Nursing - 270

HOSPITAL MEETINGS AND SOCIETIES.—Society for the Study of Inebriety—The Metropolitan Hospital - 272

### EXTRA SUPPLEMENT.—THE NURSING MIRROR.

**En Passant.**—Memorial to Nurse Coates—On Commission—Sydney Hospital—Christmas Competitions—Another's Reputation—The Society for the Employment of Women—Kensington District Nursing Association - cvii

**Ventilation, Disinfection, and Diet.** By P. CALDWELL SMITH. XIV.—Diet and Dietaries - cviii

**A Plan of Instruction in Invalid Cooking for Training Schools.** By ISABEL A. HAMPTON, Superintendent of Nurses, and Principal of the Training School of the Johns Hopkins Hospital - cix

**Sisters.** - cx

**Everybody's Opinion.**—The Matronship of Longton Cottage Hospital - cx

**Presentations** - cxi

**Appointment** - cxi

**Wants and Workers** - cxi

**For Reading to the Sick.**—"Light 'mid the encircling gloom" - cxi

**Off Duty.**—The Medicine Boy - cxii

**Notes and Queries** - cxii

